# Prevalence of cervical high-risk human papillomavirus among Zimbabwean women living with HIV

**DOI:** 10.4102/sajhivmed.v25i1.1633

**Published:** 2024-12-12

**Authors:** Tarisai Kufa, Ardele Mandiriri, Tinei Shamu, Racheal S. Dube Mandishora, Margaret J. Pascoe

**Affiliations:** 1 Newlands Clinic, Ruedi Luethy Foundation - Zimbabwe, Harare, Zimbabwe; 2 Graduate School of Health Sciences, University of Bern, Bern, Switzerland; 3 Institute of Social and Preventive Medicine, University of Bern, Bern, Switzerland; 4 Department of Laboratory Diagnostic and Investigative Sciences, Medical Microbiology Unit, University of Zimbabwe, Harare, Zimbabwe; 5 Center for Immunization and Infection Research in Cancer, Moffit Cancer Center, Florida, United States of America

**Keywords:** HPV, HIV, women, cervix, distribution

## Abstract

**Background:**

Women living with HIV (WLWH) are six times more likely to develop cervical cancer (CC). There is also an increased incidence of CC in women with optimal HIV disease control, despite immune reconstitution due to antiretroviral therapy (ART).

**Objectives:**

This study describes the prevalence and age-specific genotype distribution of hrHPV among an urban cohort of WLWH. Additionally, we report the HIV disease profile and age-specific outcomes of hrHPV DNA screening in WLWH attending routine CC screening at Newlands Clinic, Harare, between January and December 2021.

**Method:**

This was a descriptive cross-sectional design based on a retrospective review of records of WLWH who were screened for hrHPV infection. We assessed the prevalence of hrHPV infection during the study period.

**Results:**

We included data for 2745 women who had an hrHPV DNA test. The median age at the time of testing was 45 years (interquartile range [IQR]; 37–52) The median duration on HIV ART was 10.2 years (IQR: 6.2–13.3). The proportion of women with undetectable viral loads (< 50 copies/mL) was 91.2%. The prevalence of hrHPV infection was 53%. The most prevalent genotypes were human papillomavirus (HPV) 58 (11%), HPV 52 (10%), HPV 35 (10%), and HPV 16 (9%).

**Conclusion:**

Our study reports a high prevalence of HPV and hrHPV including other subtypes than 16 and 18. These results highlight the continued importance of CC screening and prophylactic HPV vaccinations among WLWH.

**What this study adds:** The study adds to the knowledge of the prevalence of hrHPV in WLWH of various ages, and highlights the prevalence of HPV genotypes other than 16 and 18. The study emphasises the significance of CC screening and HPV vaccination, especially for women with well-controlled HIV infection.

## Introduction

Persistent infection with high-risk human papillomavirus (hrHPV) is a necessary cause for cervical cancer (CC).^1^ Women living with HIV (WLWH) are six times more likely to develop CC because of their increased risk of human papillomavirus (HPV) acquisition and persistence.^2,3^ This risk remains, despite optimal HIV disease control and immune reconstitution due to effective antiretroviral therapy (ART). In women with suboptimal HIV disease control, the risk is further increased, illustrating the knowledge gaps regarding the effect of HIV disease control on the prevalence, incidence, and/or clearance of hrHPV.

It has been well established that of the oncogenic hrHPV genotypes, 16 and 18 cause 70% of CC and higher percentages of other HPV-related cancers. The prevalence and genotype- specific distribution is reported to depend on age and geographic location.^4^ Several studies have shown that hrHPV is more prevalent in younger women,^5^ and the dominant genotypes associated with CC apart from 16 to 18 in Africa and Asia are 58, 45, 35 and 52.^6^ Data from Zimbabwe report genotypes 18 and 52 as the predominant genotypes associated with CC,^7,8^ followed by 31, 33, 45, 52, and 58 which account for an additional 10% – 20%.^9^

Two key pillars in the CC elimination strategy are primary prevention of hrHPV by vaccination and secondary prevention with the timely detection and treatment of precancerous lesions.^7,10^ Three vaccines that prevent infection with disease-causing HPV have been licensed by the United States Food and Drug Administration (FDA). These are Cervarix (bivalent vaccine), which prevents infection with genotypes 16 and 18; Gardasil 4 (quadrivalent vaccine), which confers protection against genotypes 6,11, 16 and 18; and Gardasil 9 (nonavalent vaccine), which protects against genotypes 6, 11, 16, 18, 31, 33, 45, 52, 58.

Zimbabwe has one of the highest incidences of CC, with an age-standardised rate of 62 per 100 000, five times higher than the global average.^11^ In 2018, an estimated 3186 women were diagnosed with CC and 2151 died due to the disease.^4^ The national CC prevention services include a school-based HPV vaccination programme targeting girls at 10 years of age, and using the bivalent vaccine. The mainstay of secondary prevention is visual inspection of the cervix with acetic acid and cervicography (VIAC),^4^ which detects the presence of acetowhite lesions. Treatment is dependent on the size and location of the lesion, with larger lesions requiring more excisional treatment.^12^ In 2021, the World Health Organization (WHO) recommended that HPV DNA testing be implemented as the primary CC screening method,^13^ and the Ministry of Health and Child Care (MoHCC) began the process to move from VIAC to HPV DNA screening. The Newlands Clinic in Harare, Zimbabwe, acquired the CFX 96 platform and adopted HPV DNA screening for 14 high-risk genotypes in women receiving HIV care and attending for routine CC screening.

In this study we set out to describe the prevalence and age- specific genotype distribution of hrHPV among an urban cohort of WLWH. In addition, we report the HIV disease profile, age-specific outcomes and highlight factors associated with hrHPV positivity in a cohort of WLWH attending routine CC screening between January 2021 and December 2021.

## Research methods and design

### Study design

This was a descriptive cross-sectional study based on a retrospective review of records of WLWH who were routinely screened for hrHPV infection between 01 January 2021 and 31 December 2021. We described the characteristics and clinical evidence of cervical disease as defined by VIAC status, and assessed the prevalence of hrHPV infection during the study period.

### Study setting

**FIGURE 1:**
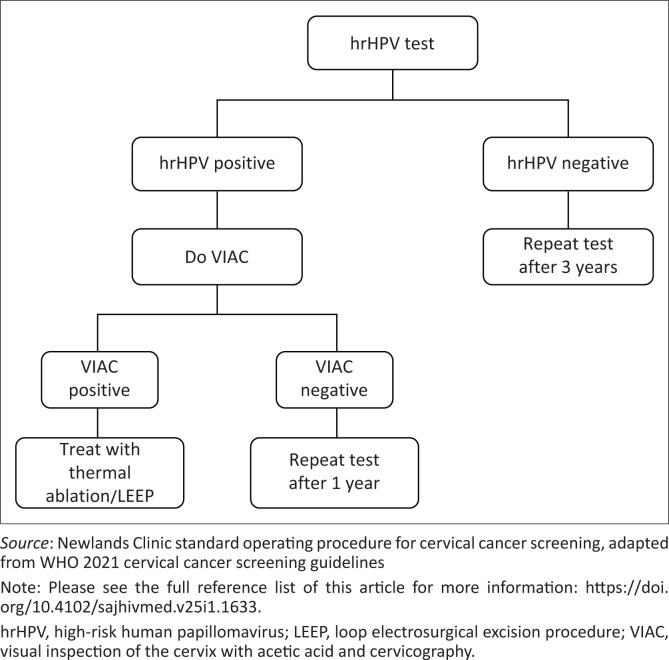
Newlands Clinic protocol for management after human papillomavirus DNA testing.

The study was conducted at Newlands Clinic, an urban HIV clinic in Harare, Zimbabwe, offering comprehensive HIV management, as described previously.^13,14^ Briefly, the Newlands Clinic was established in 2004 in partnership with the MoHCC, and provides comprehensive HIV care to approximately 8000 people living with HIV; women above the age of 25 years constitute 63%. Routine annual CC screening is a free service which is part of the comprehensive HIV care package, and all sexually active women and women and girls reporting sexual assault are invited for CC screening. In 2021, HPV DNA testing was adopted as the primary screening modality for CC. Women with a positive hrHPV test had a VIAC and were managed according to the protocol (Figure 1).^12^ Data for sexually active women 18 years and above, who had a clinician-collected cervical swab for HPV DNA polymerase chain reaction (PCR) testing, were included in this study.

### Human papillomavirus DNA sample collection and testing

A cervical swab was collected by a healthcare worker and processed in the onsite laboratory. For this purpose, eNAT^®^ swabs by COPAN Diagnostics (Murrieta, California, United States) were used. DNA extraction was performed using the magnetic bead-based CleanPrepX Kit DNA kits on a PurePrep 96 (Molgen, Veenendaal, the Netherlands). Genotyping was performed using the Allplex™ HPV HR Detection kit (Seegene, Inc., Seoul, South Korea) processed on the CFX-96 real time PCR assay (Bio Rad, Hercules, California, United States) which detects 14 hrHPV genotypes (16, 18, 31, 33, 35, 39, 45, 51, 52, 56, 58, 59, 66, 68). Results were interpreted using Seegene Viewer software (Seegene, Inc., Seoul, South Korea).

Women with a positive hrHPV DNA and a positive VIAC result were treated with thermal ablation or loop electrosurgical excision procedure (LEEP), depending on the size and site of the lesion. Thermal ablation was performed for ectocervical lesions which were present in less than 75% of the transformation zone, and LEEP was performed for endocervical lesions and lesions present in more than 75% of the transformation zone. The LEEP provided tissue specimens which were sent to an offsite laboratory for histological analysis. Cervical punch biopsies were performed in women with lesions suspicious for cancer, and the samples were sent for histological analysis. A histological diagnosis of High-grade Squamous Intraepithelial Lesion (HSIL) was defined as precancer.

### Data abstraction

Patient data for the analysis were retrieved from the clinic’s electronic medical record system known as Electronic Point of Care (eEPOC) into Microsoft Excel. To extract relevant data for the current analysis, customised queries were developed in Microsoft SQL Server Management Studio 2012, applying inclusion and exclusion criteria directly within the queries to filter for the specific patient population of interest. The results of these queries were exported to Microsoft Excel, and the data were then imported into Stata version 13.1 (StataCorp LP, College Station, Texas, United States) for further analysis. We included all eligible participants in the database who were enrolled into the cohort and had a CC screening using HPV between January 2021 and December 2021. Variables abstracted included demographics (age), HIV clinical data (CD4 count, and viral load measured within 6 months of the hrHPV test), CC screening data (HPV tests, VIAC results). The VIAC status was recorded as either positive or negative and the treatment was recorded.

### Analysis

Descriptive statistics were used to characterise patients. Categorical variables were reported as absolute numbers (*n*) and relative frequency (%), and continuous variables such as median and interquartile range (IQR) and were stratified by age. Chi-square test of association and associated odds ratios and 95% confidence interval were calculated for independent associations between categorical variable and hrHPV infection. All statistical analyses were performed using Stata version 13.1 and R Statistical Software (v4.3.1; R Core Team 2023).

### Ethical considerations

Ethical approval for the study was granted by the Medical Research Council of Zimbabwe, reference MRCZ/E/345. Individuals whose data were used in this study provided written informed consent, which allowed for the data collected during their routine care to be used for research. The study did not include minors. The data set was de-identified before being shared with the researcher and the electronic data transfer was encrypted. The data set was kept on a password-protected laptop by the researchers.

## Results

We included data for 2745 women who had an hrHPV test between January 2021 and December 2021. Data for 20 women (1%) who had a test failure that could not be repeated in the year were excluded. The remaining data for 2725 women (99%) were included in the analysis.

The median age at the time of testing was 45 years (IQR: 37–52) The median duration on ART was 10.2 years (IQR: 6.2–13.3). Thirteen women were ART naïve. At the time of screening, 99% (2696/2725) of the women had CD4 count results, 4.7% (*n* = 127/2696) with a result less than 200 cells/mm^3^. The proportion of women with undetectable viral loads (< 50 copies/mL) was 91.2%, while 6.0% had low-level viraemia (50 copies/mL –999 copies/mL) (Table 1). Viral load and CD4 count were associated with age in the univariate analysis. With every year increase in age, women were more likely to be virologically suppressed (odds ratio [OR]: 1.1; 95% CI: 1.03–1.08) and to have immunological competency (OR: 1.02 95% CI: 1.00–1.04). Nineteen women had other HPV-related anogenital disease.

**TABLE 1:** Age-stratified clinical and demographic profile of women living with HIV screened for high-risk human papillomavirus infection (Harare, January–December 2021, *n* = 2725).

Variable	Age category (years)												Total			
	18–24 (*n* = 144; 5.3%)				25–44 (*n* = 1703; 62.5%)				45+ (*n* = 878; 32.2%)							
	*n*	%	Median	IQR	*n*	%	Median	IQR	*n*	%	Median	IQR	*n*	%	Median	IQR
**Level of education**																
None/Primary	70	48.6	-	-	168	14.4	-	-	304	21.5	-	-	542	19.9	-	-
Secondary/Tertiary	74	51.4	-	-	1000	85.6	-	-	1109	78.5	-	-	2183	80.1	-	-
**CD4 count (cell/mm^3^)**																
< 200	15	10.4	-	-	62	5.3	-	-	50	3.5	-	-	127	4.7	-	-
> 200	128	89.9	-	-	1092	93.5	-	-	1349	95.5	-	-	2569	94.3	-	-
Missing	1	0.7	-	-	14	1.2	-	-	14	1.0	-	-	29	1.1	-	-
**Viral load (copies/mL)**																
< 50	116	80.6	-	-	1063	91.0	-	-	1307	92.5	-	-	2486	91.2	-	-
50–1000	16	11.1	-	-	63	5.4	-	-	84	5.9	-	-	163	6.0	-	-
> 1000	12	8.3	-	-	42	3.6	-	-	22	1.6	-	-	76	2.8	-	-
History of HIV treatment failure	3	2.1	-	-	49	4.2	-	-	56	4.0	-	-	108	4.0	-	-
Years on ART	-	-	9.5	3.3–12.0	-	-	8.1	2.5–11.5	-	-	11.7	7.8–14.8	-	-	10.2	6.2–13.3
STI history	44	30.6	-	-	413	35.4	-	-	516	36.5	-	-	973	35.7	-	-
Other HPV-related anogenital disease	13	70.0	-	-	4	20.0	-	-	3	10.0	-	-	19	0.7	-	-

ART, antiretroviral therapy; STI, sexually transmitted infection; HPV, human papillomavirus; IQR, interquartile range.

The overall prevalence of hrHPV was 1445/2725 (53%; 95% CI: 51–55). The prevalence by age category was 66% (95% CI: 58–74) in 18–24 years, 54% in 25–44 years and 51% in women 45 years and older. An increasing age category of participants was associated with a reduced prevalence of hrHPV infection (OR: 0.85, 95% CI: 0.74–0.98, *P* = 0.02). Approximately two in five women in the 18 years to 24 years category had at least two hrHPV infection genotypes (Table 2), and five women had clinical evidence of HPV-associated disease in only the anogenital area.

**TABLE 2:** High-risk human papillomavirus genotype testing outcomes by age group.

Age group (years)	Number screened	Positive hrHPV screen result		Proportion with multiple hrHPV infection	
		%	95% CI	*n*	%
18–24	144	66	58–74	61	42
25–44	1168	54	52–57	295	25
> 45	1413	51	48–53	323	23

CI, confidence interval; hrHPV, high-risk human papillomavirus.

**FIGURE 2:**
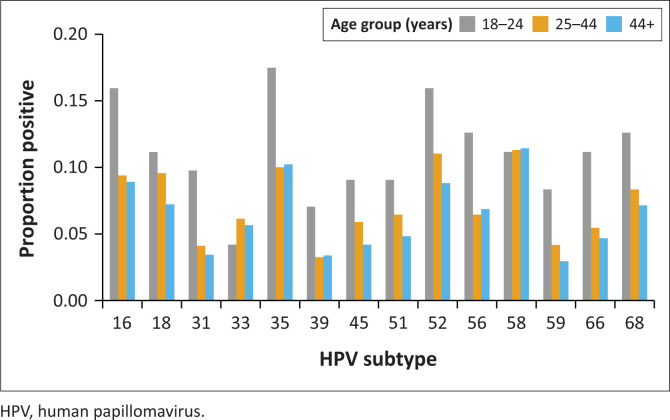
Prevalent high-risk human papillomavirus infection genotype distribution by age category.

Overall, the most prevalent genotypes were HPV 58 (11%), HPV 52 (10%), HPV 35 (10%), and HPV 16 (9%) (Figure 2). In women aged 18–24 years, the most common hrHPV genotype was HPV 35 (17%), whereas HPV 58 was the most common in women aged 25–44 years and older (11%) (Figure 2).

### Other patient factors associated with high-risk human papillomavirus infection

**FIGURE 3:**
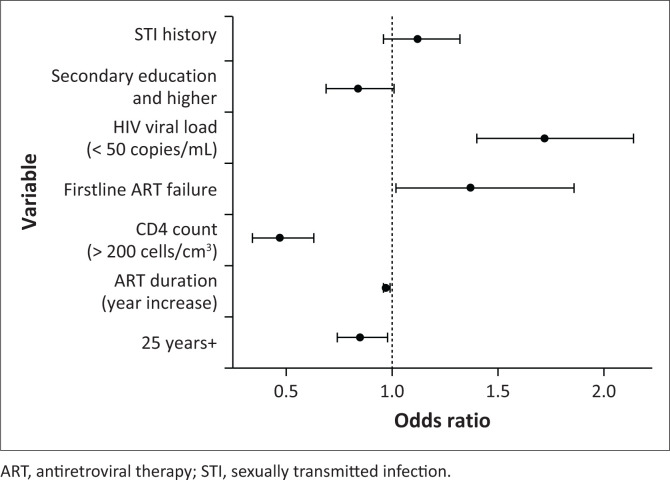
Factors associated with high-risk human papillomavirus infection in the univariate analysis.

We found evidence of association between detectable viral load and prevalent hrHPV infection. Women with a detectable viral load of > 1000 copies/mL were four times more likely to have prevalent hrHPV infection compared to those with undetectable viral loads (< 50 copies/mL) (*P* < 0.001). Similarly, lower immunological competence (≤ 200), was associated with increased odds of prevalent hrHPV infection (OR: 4.13, 95% CI: 2.29–7.44, *P* < 0.001). Treatment of STIs and a history of treatment failure on first-line ART was also associated with hrHPV infection. Figure 3 shows factors associated with hrHPV infection in the univariate analysis.

**FIGURE 4:**
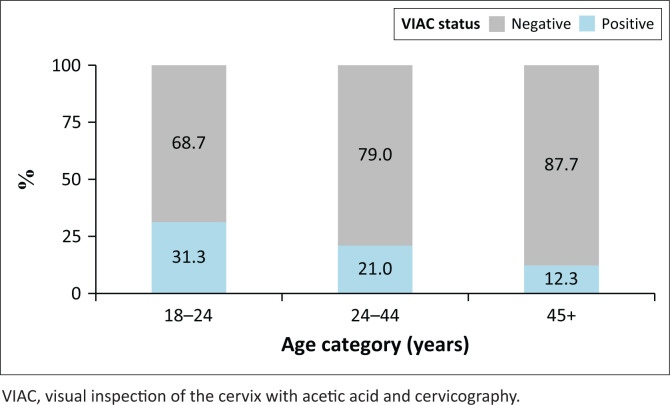
Age-stratified visual inspection of the cervix with acetic acid and cervicography status among women tested positive for high-risk human papillomavirus (*n* = 1245).

Of the 1445 patients who had a positive hrHPV test, 1245 (86%) had a VIAC done. Of these, 216 (17%) were VIAC-positive. The level of VIAC positivity was 31% (*n* = 26/83) among those aged 18–24 years, 21% (*n* = 114/544) among ages 25–44 years, and 12% (*n* = 76/618) among those aged 45 years and older (Figure 4).

### Management of cervical disease

**FIGURE 5:**
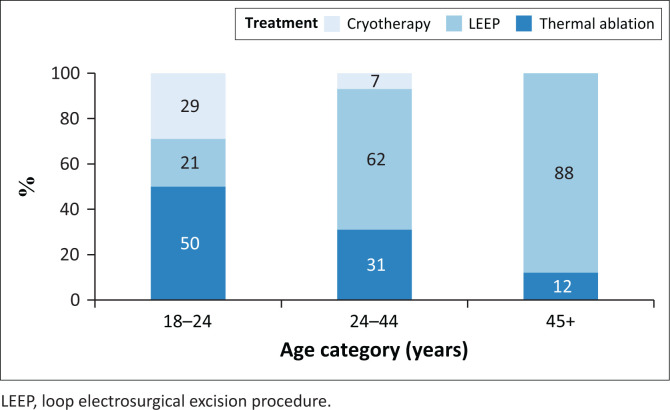
Management of cervical disease.

Among the 216 women with a positive VIAC screen, 205/216 (95%) received treatment: 136 (66%) had a LEEP, 55 (27%) had thermal ablation, and 14 (7%) had cryotherapy (Figure 5). Of the 136 treated with LEEP, 41 (30%) had histologically confirmed cervical HSIL (none in women younger than 25 years), and one woman had CC. The most prevalent cervical HPV genotypes in women with HSIL were 52 (31%), 35 (26%), and 58 (12%).

## Discussion

Our data show a high prevalence of hrHPV in WLWH, with the greatest prevalence in the 25–44-year age group. However, most of these women had optimal HIV disease control with HIV viral suppression, CD4 counts above 200 cells/mm^3^, and had been on ART for more than 5 years. Despite a high prevalence of hrHPV, only 17% of the women who tested hrHPV positive had a cervical lesion on VIAC. Among the women with HSIL, HPV genotypes 52 (31%), 35 (26%) and 58 (12%) were the most common.

The hrHPV prevalence of 53% was higher in this study of urban WLWH in the 25–45 year age group than in previously reported studies done in rural WLWH, and was four-fold higher than in women living without HIV (33% vs 13.7%).^15,16,17^ This concurs with the findings from other studies which found a higher prevalence in urban as opposed to rural settings.^18^ The participants in our study had been on ART for more than a decade, with a median ART duration of 10.4 years, and 90% had optimal HIV disease control, but the hrHPV prevalence was 53%. Data from a rural Zimbabwe and a meta-analysis study showed that being on ART for more than 2 years significantly decreased hrHPV prevalence,^7,19^ but this was not supported by the findings of this study.

The most prevalent hrHPV genotypes were 52 (10%), 35 (10%) and 58 (11%) in women with cervical precancer and overall, in the study cohort. These findings are similar to other Zimbabwean studies which showed a high prevalence of HPV genotypes 52 and 35.^7,20^ Additionally, a South African study showed an HPV 35 prevalence of 17.4% in women with cervical precancer disease and another study in Zimbabwe showed a prevalence of 26% in women with CC.^18,20^ However, this differs from European studies, which show a low prevalence of HPV 35.^20,21^ These data suggest that these HPV genotypes may indeed be more prevalent in the region. The Zimbabwean national HPV vaccination programme uses the bivalent vaccine (Cervarix), which protects against HPV genotypes 16 and 18. Several studies have shown cross protection against HPV 31, 33, 45 and, to a lesser degree, HPV35.^22,23^ This study presents further evidence that vaccine coverage should be widened to include other genotypes, for optimal prevention of CC in the region.

The prevalence of hrHPV and multiple hrHPV infections was highest among younger women and declined with increasing age. This finding is consistent with previous studies, indicating that there is an increased HPV prevalence was associated with younger age groups.^24,25^ By age stratification, HPV genotypes 35, 52 and 16 were the most common genotypes in the 18–24 year age group, while HPV genotypes 58, 35 and 16 were the most common genotypes in older women. This may indicate reduced clearance of the HPV genotypes 58, 35 and 16, as shown in other studies.^26,27^ Further longitudinal studies are required to establish the persistence and/or clearance of different hrHPV genotypes in WLWH.

In our study, VIAC screening was used as a triage test to identify women who had precancer and cancer requiring treatment. Of the women who were hrHPV positive, 17% were VIAC-positive and required treatment. The use of a high-performance initial screening test for CC results in early detection and intervention, especially in younger women.^28,29^ Furthermore, among the women who had a LEEP, only 30% had a histological diagnosis of HSIL. This is consistent with other studies which showed overtreatment rates associated with VIAC because of its low specificity.^30,31,32^

The main strengths of this study are the relatively large sample size and access to extended HPV genotyping, allowing the stratification of hrHPV prevalence by genotype. The almost complete data set allowed the analysis of a well-characterised sample and allowed the follow-up of women through the CC screening cascade using the hrHPV test in a clinical context, thus enabling the identification of important risk factors for cervical precancer and cancer.

The study had two notable limitations. Firstly, because of the cross-sectional design, we report the point prevalence of hrHPV and are unable to describe transient or persistent infection. Secondly, we could not ascertain the actual number of women with histological precancer, as women with VIAC-positive lesions who received treatment with thermal ablation did not have a histological diagnosis of the lesion as biopsies were not done prior to treatment.

## Conclusion

This study adds to the region-specific hrHPV genotype distribution data among WLWH, and the prevalence data underline the importance of CC screening and prophylactic HPV vaccination with the Gardasil vaccine which protects against nine different hrHPV types in this population. Genotypes other than 16 and 18 were prevalent in our study (HPV 35, 52, and 58), highlighting the need for access to extended genotyping and further research on the oncogenicity of these genotypes, in particular type 35. We further highlight associated risk factors in women with cervical precancer which can assist clinicians in the formulation of CC screening and management guidelines in WLWH.
